# Dissecting Water, Sanitation, and Hygiene (WaSH) to Assess Risk Factors for Cholera in Shashemene, Oromia Region, Ethiopia

**DOI:** 10.1093/cid/ciae274

**Published:** 2024-07-12

**Authors:** Dejene Hailu, Yeonji Jeon, Abel Gedefaw, Jong-Hoon Kim, Ramzi Mraidi, Tomas Getahun, Ondari D Mogeni, Edlawit Mesfin Getachew, Geun Hyeog Jang, David Mukasa, Gi Deok Pak, Deok Ryun Kim, Samuyel Ayele Abebe, Biruk Yeshitela, Moti Edosa, Yeshambel Worku Demlie, Se Eun Park, Mekonnen Teferi

**Affiliations:** Clinical, Assessment, Regulatory, Evaluation (CARE) Unit, International Vaccine Institute, Seoul, Republic of Korea; School of Public Health, Hawassa University, Hawassa, Ethiopia; Clinical, Assessment, Regulatory, Evaluation (CARE) Unit, International Vaccine Institute, Seoul, Republic of Korea; Clinical, Assessment, Regulatory, Evaluation (CARE) Unit, International Vaccine Institute, Seoul, Republic of Korea; College of Medicine and Health Sciences, Hawassa University, Hawassa, Ethiopia; Epidemiology, Public Health, Impact (EPIC) Unit, International Vaccine Institute, Seoul, Republic of Korea; Epidemiology, Public Health, Impact (EPIC) Unit, International Vaccine Institute, Seoul, Republic of Korea; Clinical Trials Directorate, Armauer Hansen Research Institute, Addis Ababa, Ethiopia; Clinical, Assessment, Regulatory, Evaluation (CARE) Unit, International Vaccine Institute, Seoul, Republic of Korea; Clinical Trials Directorate, Armauer Hansen Research Institute, Addis Ababa, Ethiopia; Epidemiology, Public Health, Impact (EPIC) Unit, International Vaccine Institute, Seoul, Republic of Korea; Epidemiology, Public Health, Impact (EPIC) Unit, International Vaccine Institute, Seoul, Republic of Korea; Epidemiology, Public Health, Impact (EPIC) Unit, International Vaccine Institute, Seoul, Republic of Korea; Epidemiology, Public Health, Impact (EPIC) Unit, International Vaccine Institute, Seoul, Republic of Korea; Statistics and Data Management Department, Armauer Hansen Research Institute, Addis Ababa, Ethiopia; Bacterial and Viral Disease Research Directorate, Armauer Hansen Research Institute, Addis Ababa, Ethiopia; Public Health Emergency Management, Ethiopia Public Health Institute, Addis Ababa, Ethiopia; Public Health Emergency Management, Ethiopia Public Health Institute, Addis Ababa, Ethiopia; Clinical, Assessment, Regulatory, Evaluation (CARE) Unit, International Vaccine Institute, Seoul, Republic of Korea; Department of Global Health and Disease Control, Yonsei University Graduate School of Public Health, Seoul, Republic of Korea; Clinical Trials Directorate, Armauer Hansen Research Institute, Addis Ababa, Ethiopia

**Keywords:** basic WaSH, WaSH risk factors, hygiene practice, Cholera, Ethiopia

## Abstract

**Background:**

Cholera outbreaks have afflicted Ethiopia, with nearly 100 000 cases and 1030 deaths reported from 2015 to 2023, emphasizing the critical need to understand water, sanitation, and hygiene (WaSH) risk factors.

**Methods:**

We conducted a cross-sectional household (HH) survey among 870 HHs in Shashemene Town and Shashemene Woreda, alongside extracting retrospective cholera case data from the Ethiopian Public Health Institute database. Relationships between WaSH and sociodemographic/economic-levels of HHs were examined. WaSH status and cholera attack rates (ARs) were described at kebele-level using geospatial mapping, and their association was statistically analyzed.

**Results:**

Access to basic drinking water, sanitation, and hygiene facilities was limited, with 67.5% (95% confidence interval, 64.4–70.6), 73.4% (70.3–76.3), and 30.3% (27.3–33.3) of HHs having access, respectively. Better WaSH practices were associated with urban residence (adjusted odds ratio, 1.7, [95% confidence interval, 1.1–2.7]), higher educational levels (2.7 [1.2–5.8]), and wealth (2.5 [1.6–4.0]). The association between cholera ARs and at least basic WaSH status was not statistically significant (multiple *R*^2^ = 0.13; *P* = .36), although localized effects were suggested for sanitation (Moran *I* = 0.22; *P* = .024).

**Conclusions:**

Addressing gaps in WaSH access and hygiene practices is crucial for reducing cholera risk. Further analyses with meaningful covariates and increased sample sizes are necessary to understand the association between cholera AR and specific WaSH components.

## INTRODUCTION

Water, sanitation, and hygiene (WaSH) are among the most important requirements for human health and well-being. However, 4.5 billion people around the world still lack access to better sanitation, and 2 billion use contaminated water sources [1]. As a result, 80% of communicable diseases are attributed to unsafe WaSH, claiming the lives of 297 000 children <5 years old each year [2, 3]. In 2016, globally 829 000 diarrheal deaths were related to unsafe WaSH [3, 4]. Diarrhea was reportedly one of the top causes of death worldwide; *Vibrio cholerae* ranked third among the causes, accounting for 107 290 deaths, of which 52 232 were in children <5 years old [4]. In 2020, consumption of contaminated water was responsible for 485 000 diarrheal deaths, and lack of safely managed sanitation caused 432 000 diarrheal deaths [3].

Over the last 2 decades (2001–2023) in Ethiopia, 215 205 cholera cases and 2355 deaths were reported, resulting in a cumulative case fatality rate (CFR) of 1.10% [5]. More recently during 2015–2023, around 99 945 cholera cases and 1030 deaths were documented, with a cumulative CFR of 1.03% [5]. Cholera CFR in Ethiopia peaked (3.13%) in 2022, and almost 30 000 cholera cases were reported nationwide in 2023 [5]). Sociodemographic and economic factors such as education level and wealth index influence access to WaSH and community water-fetching behavior [6–9], affecting cholera burden in endemic settings.

The WaSH sector encompasses availability, accessibility, and utilization pillars, which requires investigation to better identify the underlying causes hindering WaSH improvement [10]. Such data will enable more targeted WaSH interventions for better impact and resource allocation. The WaSH pillar of the Cholera Roadmap Research Agenda also questions what the most cost-effective WaSH package and coverage would be to control and eliminate cholera in various settings [11]. Numerous research endeavors have been undertaken to strengthen multisectoral cholera control interventions. These initiatives include spatiotemporal approach, Case-Area Targeted Intervention (CATI), cholera hotspot mapping or/and Priority Areas for Multisectoral Intervention (PAMI) tools, enabling target area prioritization [12–14]. Further adding to these efforts, identifying WaSH risk factors and high burden areas is important for designing adequate interventions for people living in cholera high-risk areas [15, 16].

Here, we conducted a cross-sectional household (HH) survey to better understand WaSH risk factors for cholera in Shashemene area, Ethiopia and statistically evaluated the association between cholera attack rate (AR) and WaSH status. We also mapped cholera AR and WaSH status at kebele-level to address site-specific gaps. Our findings will contribute to formulating more targeted WaSH interventions and resource allocation for better cholera control in resource-constrained settings.

## METHODS

### Study Design

A cross-sectional HH survey was conducted as part of the Ethiopia Cholera Control and Prevention (ECCP) project. The survey was rolled out in Shashemene Town (ST) and Shashemene Woreda (SW), located in Oromia region, Ethiopia, during 13–30 January 2022, before a preemptive mass oral cholera vaccine (OCV) vaccination campaign in May 2022 (Supplementary Figure 1). Both ST and SW were selected based on the national cholera hotspots mapped and included in the Ethiopian National Cholera Elimination Plan. The minimum sample size of HHs for the survey was estimated based on several assumptions. Assuming a 50% baseline in the knowledge, attitude, and practices (KAP) level in local population, the proportion of KAP in a reference group (pre-vaccination proportion) was set at 0.5, the proportion in the intervention group (post-vaccination proportion) at 0.6, and the proportion difference at 0.1, with a design effect of 2, a 95% confidence level, and a power of 0.8, resulting in an adjusted sample size of 862 HHs after accounting for a dropout rate of 10%. A total of 426 HHs in ST and 436 HHs in SW were sampled. During the actual survey, 870 HHs were interviewed (430 in ST and 440 in SW) (Supplementary Figure 2).

### Household (HH) Selection and Survey Procedure

HHs were defined as a person or a group of persons living in the same dwelling unit, sharing common food or other essentials of living and occupying a single or multiple housing infrastructures such as a single-story or multistory buildings or quarters. HHs were selected using a two-staged randomization method, taking into consideration the population density of surveillance area, inclusive of OCV vaccination area (Supplementary Table 1). The respondent to the survey was an HH head or an adult aged ≥18 years or a culturally accepted older adolescent. The respondent answered for himself/herself and on behalf of all other HH members. Written informed consent forms were obtained before the survey and an electronic data capturing system (Research Electronic Data Capture [REDCap]) was developed and used.

The survey included 3 forms (Supplementary Table 2). Form 1, on general HH information, included demographic data, general HH information, and socioeconomic- and educational-level data. Form 2, on KAP related to cholera and cholera-associated risk factors and prevention, covered WaSH and healthcare-associated factors such as vaccinations status and disease perception at HH-level. Form 3, on healthcare-seeking behavior, covered the availability and accessibility of healthcare facilities (HCFs), healthcare-seeking behavior and utilization.

Accessibility and availability of water was investigated by asking respondents about types of HH drinking water sources, distance between water source and home, water storage, and the number of persons who shared the water sources. Sanitation was assessed based on types of latrine, whether or not it was shared, its location, and whether or not the HHs practiced open defecation. Hygiene situation was investigated by collecting data on available handwashing facilities and practices. Overall WaSH practices were evaluated based on responses to several criteria, including treatment of drinking water at home, water storage method, wastewater disposal, HH waste preprocesing, use of latrines, and handwashing during critical times (i.e., before and after meals, after using latrine, and after cleaning children). The basic demographic and socioeconomic factors of the HHs were gathered to understand the relationship with basic WaSH [17].

### Retrospective Cholera Data

Data on kebele-level cholera cases in the study areas were extracted from the Ethiopian Public Health Institute (EPHI) national cholera database, covering the period from 2016 to 2020 as this database did not document any records of cases beyond this period at the time of our analysis. The national cholera case reports are typically collected through existing local public health system. Regional and national public health emergency management teams conduct cholera outbreak investigations and responses when outbreaks occur.

In Ethiopia where resources are constrained, when a suspected cholera outbreak begins, cases are initially confirmed through rapid diagnostic testing. Then, as cases escalate and *V. cholerae* stool culture–positive cases are detected from a subset of suspected cholera cases, the situation is considered a cholera outbreak, and cases are confirmed clinically. For instance, detection of *V. cholerae* in 5–10 stool cultures per outbreak sites (woreda-level) confirms a cholera outbreak, after which cases exhibiting known symptoms of cholera are diagnosed clinically [18, 19]. Thus, the cholera cases captured in the EPHI database are inclusive of clinically suspected cholera with some cases confirmed with rapid diagnostic testing or culture.

Detailed cholera surveillance, outbreak investigation and reporting system in Ethiopia are described elsewhere [5, 19]. The kebele population census estimates were obtained from the ST and SW health bureaus. Annual cholera ARs per 10 000 population at kebele-level were calculated using the annual cholera cases (numerator) and population estimates (denominator).

### WaSH Indicator Categorization

Collected HH-level WaSH status data were categorized based on the World Health Organization (WHO) and United Nations Children's Fund (UNICEF) Joint Monitoring Program (JMP) 2022 indicators [20] (Supplementary Table 3). To enable statistical evaluation of the overall WaSH practice, responses to each question were scored as either 1 (desirable response) or 0 (undesirable response), depending on the JMP guideline, to provide quantitative results. For example, if a HH head responded “using open defecation,” an undesirable response according to the JMP guideline, the response was scored as 0. After all responses were scored, they were added together to create a single composite variable. Then the 50th percentile was used as a cutoff point to categorize the scores into “better WaSH practices” or “poor WaSH practices.” “Better practice” was defined as having a WaSH practice score at the 50th percentile or above, and “poor practice” as having a score below the 50th percentile. For geospatial mapping of cholera AR and WaSH status and their association, categorization of WaSH indicators and variables was processed, and the detailed categorization used in the data analysis is described in Supplementary Table 4. The HH-level WaSH data collected were grouped by kebele to assess the kebele-level WaSH status in ST and SW.

### Data Analysis

We used SPSS software (version 26.0) for the statistical analysis of WaSH risk factors. Proxy indicators of the availability of at least basic WaSH facilities were assessed together with HH demographic and economic factors, using binary logistic regression. The assumption of goodness of fit was checked using the Hosmer and Lemeshow test [21]. Adjusted odds ratios (AORs) were used as a measure of the strength of associations, with 95% confidence intervals (CIs) and significance set at *P* < .05. We adjusted for the effect of background variables such as age, sex, place of residence, and wealth index, while identifying important predictors of access to better WaSH facilities and practices. Risk factors associated with better WaSH practice (scoring ≥50th percentile) were assessed using crude odds ratios and AORs. We used χ^2^ tests to compare the distribution of categorical variables between ST and SW and examined the association between HH WaSH status and ARs at kebele-level.

We mapped the spatial distribution of cholera AR and WaSH status at kebele-level. All shapefiles used in geospatial mapping were obtained from the openAfrica platform [22], and the mapping was conducted using QGIS 3.22.3 software. We used R 4.3.0 software for any statistical analysis to evaluate the association between WaSH and cholera AR at kebele-level. The proportion of HHs with at least basic WaSH status was treated as an independent variable, and cholera AR was used as a dependent variable. Locally estimated scatterplot smoothing (LOESS), linear regression, and Kendall τ correlation were performed to explore the correlation between WaSH status and cholera AR, and the Moran *I* statistic was computed to evaluate spatial autocorrelation between neighboring kebeles regarding WaSH components.

## RESULTS

### Availability, Accessibility, and Practice of WaSH

Approximately 95.5% of HHs (831/870) across ST and SW had improved source of drinking water (Table 1). However, only 38.6% (170/440) in SW had at least basic drinking water available, compared with 97% (417/430) in ST. More than half of HHs in ST (51.6% [222/430]) did not share drinking water source whereas 59.5% (262/440) of HHs in SW shared with more than 50 people. The distance from the drinking water source was <1000 m for the majority of HHs in ST (418/430 [97.2%]), but ≥1000 m for more than half of HHs in SW (233/440 [53.0%]).

**Table 1.** Availability and Access to Drinking Water Supply by Residence in Shashemene Town and Shashemene Woreda, 2022

Characteristic	HHs, No. (%)			*P* Value^a^
	ST (n = 430)	SW (n = 440)	Total (N = 870)	
**Source of drinking water**				
Improved	419 (97.4)	412 (93.6)	831 (95.5)	<.001
Unimproved	10 (2.3)	1 (0.2)	11 (1.3)	
Surface water	0 (0.0)	27 (6.1)	27 (3.1)	
Unknown^b^	1 (0.2)	0 (0.0)	1 (0.1)	
**Drinking water source availability** ^c^				
At least basic	417 (97.0)	170 (38.6)	587 (67.5)	<.001
Limited	7 (1.6)	206 (46.8)	213 (24.5)	
Unimproved	1 (0.2)	13 (3.0)	14 (1.6)	
Surface water	0 (0.0)	27 (6.1)	27 (3.1)	
Unknown^b^	5 (1.2)	24 (5.5)	29 (3.3)	
**No. of people sharing drinking water** **source**				
<10	107 (24.9)	74 (16.8)	181 (20.8)	<.001
10–24	37 (8.6)	6 (1.4)	43 (4.9)	
25–49	13 (3.0)	35 (8.0)	48 (5.5)	
≥50	45 (10.5)	262 (59.5)	307 (35.3)	
Not shared	222 (51.6)	61 (13.9)	283 (32.5)	
Unknown^b^	6 (1.4)	2 (0.4)	8 (0.9)	
**Distance from drinking water source**				
<1000 m	418 (97.2)	183 (41.6)	601 (69.1)	<.001
≥1000 m	7 (1.6)	233 (53.0)	240 (27.6)	
Unknown^b^	5 (1.2)	24 (5.5)	29 (3.3)	
**Distance from water source for cooking**	**(n=425)**	**(n=415)**	**(N=840)**	
<1000 m	418 (98.4)	220 (53.0)	638 (76.0)	<.001
≥1000 m	7 (1.6)	195 (47.0)	202 (24.0)	
**Distance from water source for bathing/latrine**	**(n=419)**	**(n=404)**	**(N=823)**	
<1000 m	412 (98.3)	211 (52.2)	623 (75.7)	<.001
≥1000 m	7 (1.7)	193 (47.8)	200 (24.2)	

Abbreviations: HHs, households; ST, Shashemene Town; SW, Shashemene Woreda.

The bold values refer to different total number of respondents for the variables due to missing responses.

^a^ *P* values based on χ^2^ test.

^b^If the HH's response was “No response,” “Don't know,” or “Other” with no specification, the HH was categorized as “Unknown.” “No response” was the answer that could be chosen by when respondents did not want to answer the question. There were no missing values; all 870 surveyed HHs responded to the relevant questions to determine drinking water status.

^c^Assessed based on World Health Organization/Joint Monitoring Program indicators.

Approximately 83.0% of HHs (722/870) in the Shashemene area (ST and SW) had an improved type of latrine facility, and 77.2% of HHs (332/430) in ST and 69.8% (307/440) in SW had at least basic sanitation facility available (Table 2). Still, 16.1% of HHs (71/440) in SW and 3.0% (13/430) in ST practiced open defecation, and 16.7% of HHs (72/430) in ST had limited sanitation available. Only 30.3% of the HHs (264/870) in the Shashemene area reported having access to basic hygiene facilities; 43.7% (188/430) in ST and 17.3% (76/440) in SW (Table 3). More HH members in ST cleaned their hands after urination or defecation with water and soap (58.8% [253/430]) compared to SW (27.5% [121/440]). The majority of HHs in SW showed poor WaSH practice (63.2% [278/440]).

**Table 2.** Availability and Access to Sanitation Facilities by Residence in Shashemene Town and Shashemene Woreda, 2022

Characteristic	HHs, No. (%)			*P* Value^a^
	ST (n = 430)	SW (n = 440)	Total (N = 870)	
**Type of latrine facility**				
Improved	407 (94.7)	315 (71.6)	722 (83.0)	<.001
Unimproved	12 (2.8)	72 (16.4)	84 (9.7)	
No latrine	11 (2.6)	53 (12.0)	64 (7.4)	
Don't know	0 (0.0)	0 (0.0)	0 (0.0)	
No response	0 (0.0)	0 (0.0)	0 (0.0)	
**Sanitation** **availability**^b^				
At least basic	332 (77.2)	307 (69.8)	639 (73.4)	<.001
Limited	72 (16.7)	97 (22.0)	81 (9.3)	
Unimproved	7 (1.6)	38 (8.6)	45 (5.2)	
Open defecation	13 (3.0)	71 (16.1)	84 (9.7)	
Unknown^c^	6 (1.4)	15 (3.4)	21 (2.4)	
**No. of people sharing latrine on neighborhood property**				
<10	21 (4.9)	6 (1.4)	27 (3.1)	.12
10–24	26 (6.0)	0 (0.0)	26 (3.0)	
25–49	2 (0.5)	0 (0.0)	2 (0.2)	
≥50	7 (1.6)	2 (0.5)	9 (1.0)	
No response	1 (0.2)	0 (0.0)	1 (0.1)	
Unknown	0 (0.0)	0 (0.0)	0 (0.0)	
**No. of people sharing community latrine**				
<10	12 (2.8)	6 (1.4)	18 (2.1)	.38
10–24	2 (0.5)	0 (0.0)	2 (0.2)	
25–49	0 (0.0)	1 (0.2)	1 (0.1)	
≥50	5 (1.2)	2 (0.5)	7 (0.8)	
**Distance of disposal site for wastewater** **from human waste**				
≥1 to <5 m	48 (11.2)	38 (8.6)	86 (9.9)	<.001
≥5 to <25 m	179 (41.6)	167 (38.0)	346 (39.8)	
≥25 to <50 m	56 (13.0)	78 (17.7)	134 (15.4)	
≥50 to 100 m	59 (13.7)	47 (10.7)	106 (12.2)	
≥100 to <500 m	37 (8.6)	16 (3.6)	53 (6.1)	
≥500 to 1000 m	18 (4.2)	2 (0.5)	20 (2.3)	
≥1000 m	11 (2.6)	26 (5.9)	37 (4.3)	
Unknown	7 (1.6)	11 (2.5)	18 (2.1)	
No response	15 (3.5)	55 (12.5)	70 (8.0)	

Abbreviations: HHs, households; ST, Shashemene Town; SW, Shashemene Woreda.

^a^ *P* values based on χ^2^ test.

^b^Assessed based on World Health Organization/Joint Monitoring Program indicators.

^c^If the HH's response was “No response,” “Don't know,” or “Other” with no specification, the HH was categorized as “unknown.” “No response” was the answer that could be chosen when respondents did not want to answer the question. There were no missing values; all 870 surveyed HHs responded to the relevant questions to determine sanitation status.

**Table 3.** Availability and Access to Hygiene Facility and Practice by Residence in Shashemene Town and Shashemene Woreda, 2022

Characteristic	HHs, No. (%)			*P* Value^a^
	ST (n = 430)	SW (n = 440)	Total (N = 870)	
**Hygiene facility availability** ^b^				
At least basic	188 (43.7)	76 (17.3)	264 (30.3)	<.001
Limited	220 (51.2)	294 (66.8)	514 (59.1)	
No facility	15 (3.5)	30 (6.8)	45 (5.2)	
Unknown^c^	7 (1.6)	40 (9.1)	47 (5.4)	
**HH members clean their hands after urination or defecation**				
With water and soap	253 (58.8)	121 (27.5)	374 (43.0)	<.001
With water only	165 (38.4)	256 (58.2)	421 (48.4)	
With other materials^d^	2 (0.5)	11 (2.5)	13 (1.5)	
Don't wash, not needed	2 (0.5)	20 (4.5)	22 (2.5)	
Don't wash, nothing to clean	7 (1.6)	10 (2.3)	17 (2.0)	
Don't know	1 (0.2)	16 (3.6)	17 (2.0)	
No response	0 (0.2)	6 (1.4)	6 (0.7)	
**Overall WaSH practice**				
Better^e^	301 (70.0)	162 (36.8)	463 (53.2)	<.001
Poor^f^	129 (30.0)	278 (63.2)	407 (46.8)	

Abbreviations: HHs, household; ST, Shashemene Town; SW, Shashemene Woreda; WaSH, water, sanitation, and hygiene.

^a^ *P* values based on χ^2^ test.

^b^Assessed based on World Health Organization/Joint Monitoring Program indicators.

^c^If the HH's response was “No response,” “Don't know,” or “Other” with no specification, the HH was categorized as “unknown.” “No response” was the answer that could be chosen when respondents did not want to answer the question. There were no missing values; all 870 surveyed HHs responded to the relevant questions to determine hygiene status.

^d^Other materials included leaf, straw, grass, sand, ash, and cloth/fabric.

^e^“Better” was defined as scoring at the 50th percentile or above.

^f^“Poor” was defined as scoring below the 50th percentile.

The status of handwashing practices is more elaborated in Supplementary Figure 3. On handwashing practices before and after defecation, HHs in both ST and SW reported more handwashing after than before defecation, and more HHs in ST than in SW practiced at least basic handwashing after defecation (59.0% vs 28.9%, respectively). Most HHs (85.0% [739/869]) in the Shashemene area did not treat water for drinking (Table 4), which was similar regardless of urban or rural settings. Domestic wastewater and human waste/excreta were disposed in public open fields in ST (19.3% [83/430] for wastewater and 27.0% [116/430] for human waste/excreta) and in private open fields in SW (43.7% [192/439] and 44.4% [195/439], respectively). The majority of HHs in the Shashemene area (64.0% [556/869]) did not preprocess domestic waste. In 78.1% of HHs (679/869) in the Shashemene area, latrines were used by all HH members, with no restriction.

**Table 4.** Practice of Households Regarding Water, Sanitation, and Hygiene by Residence in Shashemene Town and Shashemene Woreda, 2022

Characteristic	HHs, No. (%)			*P* Value^a^
	ST (n = 430)	SW (n = 439)	Total (N = 869)	
**Water treated for drinking**				
No, not at all	368 (85.6)	371 (84.5)	739 (85.0)	.86
Yes, for sick/children	7 (1.6)	8 (1.8)	15 (1.7)	
Yes	48 (11.2)	49 (11.2)	97 (11.2)	
Yes, during dry/rainy season	1 (0.2)	2 (0.5)	3 (0.3)	
Don't know	3 (0.7)	7 (1.6)	10 (1.2)	
No response	3 (0.7)	2 (0.5)	5 (0.6)	
**Types of water storage** ^b^				
Water tank (covered)	282 (65.6)	93 (21.2)	375 (43.2)	<.001
Bottle (covered)	243 (56.5)	252 (57.4)	495 (57.0)	.79
Clay pot (covered)	18 (4.2)	41 (9.3)	59 (6.8)	.003
Bucket (covered)	214 (49.8)	204 (46.5)	418 (48.1)	.33
Bowl (covered)	44 (10.2)	59 (13.4)	103 (11.9)	.14
Wooden pot (covered)	3 (0.7)	0 (0.0)	3 (0.3)	.08
Not stored	1 (0.2)	2 (0.5)	3 (0.3)	.58
Skin vessels	4 (0.9)	22 (5.0)	26 (3.0)	<.001
**Disposal site of wastewater from cooking** ^b^				
Open field (private)	89 (20.7)	256 (58.3)	345 (39.7)	<.001
Open field (public)	131 (30.5)	30 (6.8)	161 (18.5)	
In a hole (private)	78 (18.1)	70 (15.9)	148 (17.0)	
In a hole (public)	25 (5.8)	34 (7.7)	59 (6.8)	
Pour in surface water	25 (5.8)	8 (1.8)	33 (3.8)	
Pour into a septic tank	60 (14.0)	3 (0.7)	63 (7.2)	
Other^c^	119 (27.7)	1 (0.2)	120 (13.8)	
Don't know/No response	11 (2.6)	37 (8.4)	48 (5.5)	
**Disposal site of domestic wastewater**				
Open field (private)	35 (8.1)	192 (43.7)	227 (26.1)	<.001
Open field (public)	83 (19.3)	30 (6.8)	113 (13.0)	
In a hole (private)	48 (11.2)	33 (7.5)	81 (9.3)	
In a hole (public)	12 (2.8)	30 (6.8)	42 (4.8)	
Pour in surface water	12 (2.8)	6 (1.4)	18 (2.1)	
Pour into a septic tank	33 (7.7)	2 (0.5)	35 (4.0)	
Other^c^	67 (15.6)	6 (1.4)	73 (8.4)	
Don't know/no response	140 (32.6)	140 (31.9)	280 (32.2)	
**Disposal site of human waste/excreta**				
Open field (private)	71 (16.5)	195 (44.4)	266 (30.6)	<.001
Open field (public)	116 (27.0)	37 (8.4)	153 (17.6)	
In a hole (private)	71 (16.5)	82 (18.7)	153 (17.6)	
In a hole (public)	20 (4.7)	27 (6.2)	47 (5.4)	
Pour into surface water	25 (5.8)	6 (1.4)	31 (3.6)	
Pour into a septic tank	101 (23.5)	39 (8.9)	140 (16.1)	
Other^d^	1 (0.2)	0 (0.0)	1 (0.1)	
Don't know/no response	25 (5.8)	53 (12.1)	78 (9.0)	
**Preprocess domestic waste** ^b^				
Yes, burn	135 (31.4)	99 (22.6)	234 (26.9)	.003
Yes, sort/separate	122 (28.4)	101 (23.0)	223 (25.7)	.07
Yes, others	4 (0.9)	0 (0.0)	4 (0.5)	.04
No	260 (60.5)	296 (67.4)	556 (64.0)	.03
Don't know	4 (0.9)	1 (0.2)	5 (0.6)	.17
No response	2 (0.5)	1 (0.2)	3 (0.3)	.62
**Use of the latrine by HH members** ^b^				
Children/infants	46 (10.7)	44 (10.0)	90 (10.4)	.74
Sick people	33 (7.7)	1 (0.2)	34 (3.9)	<.001
Senior/elderly members	14 (3.3)	1 (0.2)	15 (1.7)	<.001
No, limited access	6 (1.4)	2 (0.5)	8 (0.9)	.15
No, too far away	1 (0.2)	1 (0.2)	2 (0.2)	.99
No, bad condition	6 (1.4)	73 (16.6)	79 (9.1)	<.001
Yes, all members	356 (82.8)	323 (73.6)	679 (78.1)	<.001
Other^e^	4 (0.9)	14 (3.2)	18 (2.1)	.02
Don't know	10 (2.3)	22 (5.0)	32 (3.7)	.04
No response	1 (0.2)	3 (0.7)	4 (0.5)	.33

Abbreviations: HH, households; ST, Shashemene Town; SW, Shashemene Woreda.

^a^ *P* values based on χ^2^ test.

^b^Measured on multiple responses. One person could give more than a single answer, and therefore each row has its respective *P* value.

^c^Other responses included toilet and hole near home.

^d^Other responses included collect and store in a sack.

^e^Other responses included no latrine or not applicable.

### Underlying Factors Associated With WaSH Practice

Overall, the proportion of HHs with better WaSH practice was 53.2% (463/870 [95% CI: 49.9–56.5]), largely due to better WaSH practice in ST (70.0% [301/430]) than in SW (36.8% [162/440]) (Table 5). Better WaSH practices were 1.7 times more likely in urban (ST) than in rural (SW) settings (AOR, 1.7 [95% CI: 1.1–2.7]). The wealth index of HH and the educational level of the HH head were positively correlated with better WaSH practices. Better WaSH practices were 2.5 (95% CI: 1.6–4.0) or 1.6 (1.1–2.4) times more likely in HHs with high or middle wealth indexes, respectively. HHs with HH heads who had completed up to tertiary education were 2.7 times more likely to have better WaSH practices (AOR, 2.7 [95% CI: 1.2–5.6]).

**Table 5.** Factors Associated With Better Water, Sanitation, and Hygiene Practices in Shashemene Town and Shashemene Woreda, 2022

Characteristics	WaSH Practice, No. (%) of HHs^a^		COR^b^ (95% CI)	AOR^c^ (95% CI)
	Better	Poor		
**Residence**				
Shashemene Town	301 (65.0)	129 (31.8)	3.99 (3.0–5.3)	1.70 (1.07–2.72)
Shashemene Woreda	162 (35.0)	277 (68.2)	1.0	1.0
**Family size**				
<5	232 (50.1)	165 (40.5)	0.85 (.42–1.71)	0.43 (.18–1.01)
5–9	208 (44.9)	228 (56.0)	0.55 (.27–1.10)	0.39 (.17–.91)
≥10	23 (5.0)	14 (3.4)	1.0	1.0
**Educational level of HH head**				
Cannot read and write	25 (5.5)	60 (14.8)	1.0	1.0
Primary complete	206 (45.5)	254 (62.7)	1.94 (1.17–3.21)	1.44 (.78–2.62)
Secondary complete	110 (24.3)	63 (15.6)	4.19 (2.39–7.33)	1.927 (1.06–4.05)
Tertiary complete	112 (24.7)	28 (6.9)	9.60 (5.14–17.91)	2.68 (1.24–5.78)
**Wealth index** ^d^				
Low	144 (31.1)	223 (55.2)	1.0	1.0
Middle	127 (27.4)	115 (28.5)	1.71 (1.23–2.37)	1.59 (1.05–2.41)
High	192 (41.5)	66 (16.3)	4.50 (3.17–6.38)	2.50 (1.58–3.97)
**Availability** **of** **drinking water source**				
Accessible	359 (80.1)	242 (61.6)	2.51 (1.85–3.42)	0.46 (.27–.76)
Inaccessible	89 (19.9)	151 (38.4)	1.0	1.0
**No. of people sharing water source**				
<10	131 (28.3)	50 (12.3)	1.0	1.0
10–24	19 (4.1)	24 (5.9)	0.30 (.15–.59)	0.24 (.11–.52)
25–49	31 (6.7)	17 (4.2)	0.69 (.35–1.37)	0.69 (.32–1.46)
≥50	55 (11.9)	253 (62.3)	0.08 (.05–.13)	0.07 (.04–.13)
Not shared	224 (48.4)	59 (14.5)	1.45 (.94–2.24)	1.05 (.63–1.76)
Unknown	3 (0.6)	3 (0.7)	0.38 (.07–1.95)	0.31 (.04–2.10)

Abbreviations: AOR, adjusted odds ratio; CI, confidence interval; COR, crude odds ratio; HH, household; WaSH, water, sanitation, and hygiene

^a^“Better” was defined as scoring at the 50th percentile or above; “Poor,” as scoring below the 50th percentile. The numbers of respondent differed for different characteristics because of missing responses; therefore, column totals are not provided.

^b^Unadjusted measure of strength of association between each covariate and better practice.

^c^Adjusted measure of strength of association between set of covariates (characteristics) and better practice.

^d^The wealth index was constructed from a composite variable and categorized as low for scores below the 50th percentile, middle for scores above the 50th and below the 75th percentile, and high for scores at the 75th percentile or above.

### Factors Associated With Basic Drinking Water Supply, Sanitation, and Hygiene

High or middle wealth index (AOR, 2.7 [95% CI: 1.5–4.9] and 1.8 [1.1–2.8], respectively) and urban setting (53.9 [25.6–113.5]) were positively associated with HH access to basic drinking water. Overall, wealth index had a stronger positive association with basic sanitation (AOR [95% CI] for high and middle wealth index, 6.8 [3.6–12.8] and 4.2 [2.6–6.7], respectively) than basic drinking water (2.7 [1.5–4.9] and 1.8 [1.1–2.8]) and basic hygiene (2.8 [1.7–4.9] and 1.2 [.7–2.0]). Urban residence (AOR, 7.9 [95% CI: 4.2–14.9]), number of people sharing water sources (2.6 [1.3–4.8]), and high wealth index (2.8 [1.7–4.9]) were important factors that improved HH access to at least basic hygiene facilities (Table 6). Female sex (AOR, 0.5 [95% CI: .3–.8]), urban residence (0.4 [.2–.6]), and sharing drinking water sources reduced the odds of access to basic sanitation.

**Table 6.** Household Characteristics Associated With Basic Water, Sanitation, and Hygiene Status in Shashemene Town and Shashemene Woreda, 2022

Characteristic	AOR (95% CI)		
	Basic Drinking Water Source	Basic Sanitation	Basic Hygiene
**Sex of respondent**			
Male	1.0	1.0	1.0
Female	1.32 (.86–2.03)	0.52 (.33–.81)^a^	0.65 (.42–.99)^a^
**Residence**			
Shashemene Town	53.93 (25.64–113.46)^a^	0.35 (.21–.63)^a^	7.94 (4.24–14.85)^a^
Shashemene Woreda	1.0	1.0	…
**Educational level of HH head**			
Cannot read and write	1.0	1.0	1.0
Primary complete	0.93 (.49–1.74)	2.01 (1.11–3.65)^a^	0.56 (.29–1.08)
Secondary complete	1.55 (.73–3.29)	1.85 (.88–3.88)	0.57 (.27–1.19)
Tertiary complete	2.60 (.91–7.45)	1.30 (.55–3.07)	0.20 (.08–.46)^a^
**Distance from water source**			
<1000 m	NE^b^	1.10 (.67–1.79)	1.59 (.79–3.19)
≥1000 m		1.0	1.0
**No. of people sharing drinking water**			
<10	NE^b^	0.09 (.04–.22)^a^	0.97 (.54–1.72)
10–49		0.05 (.02–.14)^a^	1.24 (.61–2.52)
≥50		0.02 (.01–.06)^a^	2.55 (1.34–4.84)^a^
Not shared		1.0	1.0
**Respondent age (in years)**	0.99 (.97–1.01)	1.00 (.98–1.01)	0.99 (.97–1.00)
**No. of people in HH**			
<5	1.43 (.58–3.56)	0.57 (.21–1.57)	0.53 (.21–1.33)
5–10	1.49 (.62–3.61)	0.66 (.24–1.81)	0.41 (.16–1.03)
≥10	1.0	1.0	1.0
**Wealth index** ^c^			
Low	1.0	1.0	1.0
Middle	1.78 (1.12–2.83)^a^	4.21 (2.64–6.71)^a^	1.18 (.71–1.98)
High	2.72 (1.51–4.92)^a^	6.78 (3.58–12.84)^a^	2.84 (1.65–4.87)^a^
**Know the importance of proper waste disposal**			
Yes	0.73 (.45–1.21)	2.04 (1.20–3.47)^a^	1.92 (.95–3.85)
No	1.0	1.0	1.0

Abbreviations: AOR, adjusted odds ratio; CI, confidence interval; HH, household; NE, not estimated.

^a^Significant at α = .05.

^b^Not estimated because the variables were used in the computation of the outcome as composite variable.

^c^The wealth index was constructed from a composite variable and categorized as low for scores below the 50th percentile, middle for scores above the 50th and below the 75th percentile, and high for scores at the 75th percentile or above.

### Cholera Attack Rate and WaSH Status at Kebele-Level

Kebele-level annual and mean cholera ARs per 10 000 population in ST and SW between 2016 and 2020 are described in Supplementary Figures 4 and 5. Cholera cases were persistently reported in Shashamene area except in 2018. ST showed the highest cholera AR in 2016. SW recorded higher cholera AR than ST in general. Among study kebeles in SW, Alelu Iluu had the highest AR during the five-year data analysis period, followed by Bute Filicha and Faji Goba, and no case was reported from Bura Borama. In 2017, cholera cases were reported only from kebeles in the Faji Gole cluster, neighboring ST. In general, the mean cholera AR was <6% in both ST and SW, but Alelu Iluu, Bute Filicha, and Faji Goba had higher ARs than the other kebeles. Supplementary Figure 6 shows the proportion of HHs with at least basic WaSH status in Shashemene area at kebele-level. All study kebeles in ST had better drinking water source than sanitation and hygiene status. In ST, Alelu had better WaSH status than the other kebeles, whereas Arada showed the lowest level of WaSH. In SW, four kebeles showed no HH with at least basic water source and hygiene. Less than 60% of HHs in all kebeles in Shashamene, except Abosto and Awasho in ST, practiced at least basic hygiene.

### Association Between Cholera Attack Rateand WaSH Status

**Figure 1.**
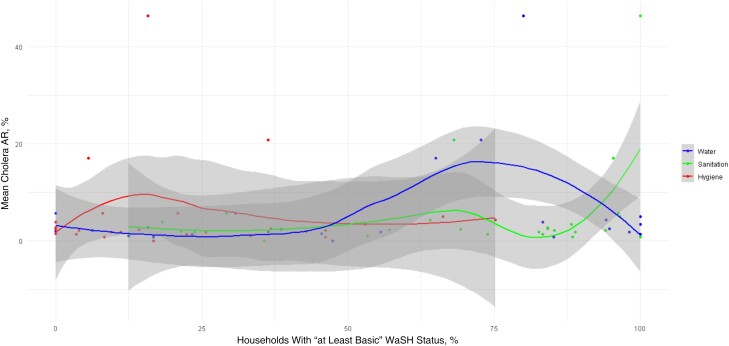
Estimated association between mean cholera attack rate (AR) from 2016 to 2020 and water, sanitation, and hygiene (WaSH) status in study kebeles in Shashemene Town and Shashemene Woreda. The association is shown for three components of WaSH with “at least basic” status: drinking water source, sanitation, and hygiene. Each line represents LOESS (locally estimated scatterplot smoothing) estimates of association between mean cholera AR and status of respective WaSH components, with nonparametric 95% confidence intervals (*gray shaded areas*). Each dot represents the surveyed kebele. The x-axis represents the proportion of households with “at least basic” WaSH status by kebele, and the y-axis, mean cholera AR.

The result of LOESS analysis, shown in Figure 1, suggests that the association between WaSH components and cholera AR was not straightforward and may involve more complex dynamics than a simple linear association. Cholera AR decreased when more than 75% of HHs at kebele had at least basic water status. However, such negative correlation was not shown when over 80% of HHs at kebele had at least basic sanitation. This controversial finding warrants further investigations to understand any underlying causes or confounding factors. Our linear regression analysis did not find a significant association between the mean cholera AR and at least basic water (*P* = .24), sanitation (*P* = .24), or hygiene (*P* = .40). The model accounted for approximately 13.3% of the variability in the mean cholera AR (multiple *R*^2^ = 0.1332), but the overall model fit was not statistically significant (*F* statistic = 1.127; *P* = .36).

Kendall τ correlation coefficients between each WaSH component and most recent cholera AR (2020) indicated very weak positive correlation that was not statistically significant (water [vs 2020 AR], Kendall τ = 0.06528422 and *P* = .65; sanitation, Kendall τ = 0.1969474 and *P* = .17; hygiene, Kendall τ = 0.2245212 and *P* = .12). The Moran *I* statistic indicated no significant spatial autocorrelation for most WaSH components (water, Moran *I* = 0.0998 and *P* = .14; hygiene, *I* = −0.1447 and *P* = .76), with the exception of a borderline significant value for sanitation (*I* = 0.2223 and *P* = .024), hinting at potential localized effects.

## DISCUSSION

Our study results showed that accessibility and availability of at least basic WaSH facilities and desirable WaSH practices were significantly lower among rural populations than urban. All study kebeles in ST had access to at least basic drinking water in >80% of HHs, while two-thirds of kebeles in SW had <60% of HHs with such access. Drinking water sources were relatively more accessible and available than sanitation and hygiene. Urban settings, HH wealth index, and the HH head's educational level were positively associated with better WaSH practice. Cholera cases were detected in both ST and SW, except in 2018, but SW had a relatively higher cholera AR than ST. A statistically significant association between cholera AR and WaSH status could not be found, but the findings imply the necessity of further spatiotemporal analysis.

In Shashemene area, the availability of at least basic sanitation facilities was about 73.4%, consistent with findings from the Wolaita Sodo woreda (75.9%) in southern Ethiopia [23], rural Tigray (68.4%), and northern Ethiopia [24] and higher than findings from the Amhara Region (59.8%) [25], southern Ethiopia (27.3%) [8], Arsi-Negele (45%) and central Ethiopia [26], and the national figure (69%) [27]. However, the availability of at least basic water and hygiene facilities in SW calls for further public health and development actions, including water supply improvement that could impact hygiene practices. Urban residents tend to have better access to WaSH facilities in Shashemene, similar to other studies in Nepal [28], Benin [29], and Ethiopia [29–31]. Local residents in Shashemene had a fairly good understanding on how to prevent cholera, but outbreaks have persistently occurred [17]. This reiterates the need for imminent investment in WaSH infrastructures.

In our geospatial visualization of cholera AR, three kebeles in Faji Gole cluster of SW were noticeable with high cholera AR every year. This cluster is located on the downward slope of ST, often receiving urban runoff during the rainy seasons. Two small streams crossing ST are likely to be contaminated with human, animal, industrial waste, and sewage water released from a nearby prison. It signifies possible environmental contamination in the neighborhood, increasing the risk of cholera transmission. Geospatial description of cholera epidemiology can be a worthwhile method when comparing interregional differences [16]. For instance, our visual maps on kebele-level WaSH status demonstrated a distinct inequality between urban and rural communities. All study kebeles in SW had <60% of HHs practicing at least basic hygiene. Other studies also described undesirable WaSH condition in rural areas [32, 33]. A study in India showed the geographical inequalities in WaSH infrastructures, signaling the need for “spatially optimized policy” [34]. To investigate the relationship between at least basic WaSH and cholera AR regardless of settings, we evaluated this at the kebele-level. Statistically insignificant association was found, but the Moran I statistic hinted at potential localized effects in sanitation status. We combined WaSH variables to categorize each WaSH component’s “at least basic” status, which may have also affected our results. Segregating each WaSH variable such as distance to water sources, types of sanitation facilities, or hygiene practices is suggested for further analysis.

Our study has some limitations. First, we did not collect data on the latrine facilities' cleanliness, individual water consumption rates, or functionality of water supplies, which are considered important WaSH factors. Some variables such as handwashing practices were also evaluated based on interviews rather than observation of actual practices. Second, there was a time discrepancy between the cholera AR and WaSH data used in the analysis. The HH-level WaSH data were collected in January 2022 whereas the cholera AR were retrospectively collected covering the period from 2016 to 2020. In ST and SW, cholera case was not documented in the EPHI database between 2021 and 2023 at the time of this data analysis. This is an under-/delayed reporting since cholera cases are captured in our sentinel surveillance network during 2022 and 2023. Further, a preemptive OCV mass vaccination campaign was conducted in our study areas in May 2022. Considering the potential impact of this vaccination on cholera AR, the association analysis between WaSH and cholera AR in this article did not include the latest cholera incidence in 2022 and 2023. Third, grouping cholera AR and WaSH status into kebele-level resulted in suboptimal sample numbers, contributing to lower power in the analysis. Kebele-level cholera AR data was the smallest administrative level available, and WaSH status was adjusted accordingly at kebele-level.

However, this is the first study done in Shashemene dissecting WaSH risk factors for cholera and mapping cholera AR and WaSH status at kebele-level. We demonstrated the need for strengthening WaSH infrastructure and hygiene practices. This is critical in the time of cholera vaccine shortage and frequent natural disasters and cholera outbreaks [35–37]. The Global Task Force on Cholera Control guidance on WaSH suggests prioritizing resources for cholera high-risk regions [38, 39]. Our study outcomes advocate for better universal WaSH coverage in Ethiopia to complement the use of OCV and other multisectoral cholera elimination strategies.

## CONCLUSION

This study demonstrated at least basic WaSH status at kebele-level in Shashemene area. Much attention is required to improve overall hygiene practices and WaSH facilities, especially in rural communities. Geospatial mapping of WaSH and cholera AR proved to be a useful tool to interrogate the inequality of WaSH pillars at the lowest public administrative units of Ethiopia.

## Supplementary Data

Supplementary materials are available at *Clinical Infectious Diseases* online. Consisting of data provided by the authors to benefit the reader, the posted materials are not copyedited and are the sole responsibility of the authors, so questions or comments should be addressed to the corresponding author.

## Supplementary Material

ciae274_Supplementary_Data

## Notes

***Acknowledgments.*** The authors acknowledge all the community survey team members, including the Research Assistants, Health Extension Workers, and local government officials in Shashemene Town and Shashemene Woreda and the Oromia Region Health Bureau for their active engagement and support of this survey and ECCP project. We thank the local communities for their consent to take part in the survey. We also extend appreciation to our research partners and dedicated staff at the ECCP study network in Ethiopia. Finally, we thank Bo-Sung Kim and Hae Kyung Cho for project administrative support.

***Author contributions.*** S. E. P. and M. T. conceptualized the overall study design of the ECCP project. Microplanning of the community survey was put together by D. H., under the supervision of S. E. P. and M. T. and in discussion with ECCP team members. A. G., T. G., E. M. G., and B. Y. implemented the survey, together with survey teams in Shashemene Town and Shashemene Woreda. D. H., Y. J., A. G., T. G., O. D. M., E. M. G., D. M., and S. A. A. conducted database review, including error checks and corrections. D. H. and Y. J. drafted the manuscript, in discussion with J. H. K., R. M., D. R. K., and S. E. P. All authors read and provided feedback and approved the final draft.

***Data availability.*** All data relevant to the study are included in this article.

***Financial support*.** This study was supported by the Korea Support Committee for International Vaccine Institute (CHMTD05083-010), LG Electronics (CHMTD05083-020), and Community Chest of Korea (CHMTD05083-030). The International Vaccine Institute (IVI) acknowledges its donors, including the government of the Republic of Korea and the Swedish International Development Cooperation Agency.

***Supplement sponsorship.*** This article appears as part of the supplement “Ethiopia Cholera Control and Prevention (ECCP): Evidence-Generation Towards Global Roadmap to Ending Cholera,” sponsored by the IVI.
