# Supplementary material for: Dissecting Water, Sanitation, and Hygiene (WaSH) to Assess Risk Factors for Cholera in Shashemene, Oromia Region, Ethiopia
Source: Clin Infect Dis. 2024 Jul 12;79(Suppl 1):S53–62. doi: 10.1093/cid/ciae274 (PMC11244206; doi:10.1093/cid/ciae274)
Supplement: ciae274_Supplementary_Data [file ciae274_supplementary_data.zip › CID-120385R2_supp materials R2.docx]

**Supplementary Table 1-1. Household selection for community survey in Shashemene Town**

| **Name of PHCU^1^ and Kebeles** | | **Number of HHs^2^ in 2021** | **Number (%) of HHs^2^ selected** |
| --- | --- | --- | --- |
| **Abosto** | | | |
|  | Gelgele K1&2 | 775 | 25 (3%) |
|  | Demitu K6 | 637 | 20 (3%) |
|  | Demitu K10 | 248 | 8 (3%) |
|  | Garite K5 | 543 | 17 (3%) |
|  | Garite K7 | 588 | 19 (3%) |
|  | Mehari K3 | 782 | 25 (3%) |
|  | Bashu K9 | 64 | 2 (3%) |
|  | Besha K4 | 576 | 18 (3%) |
|  | **Total** | **4,213** | **134 (3%)** |
| **Allelu** | | | |
|  | Arabe | 577 | 10 (2%) |
|  | Mash Ak K6 | 608 | 11 (2%) |
|  | Sr Shage Hussen | 308 | 5 (2%) |
|  | Shamene | 601 | 10 (2%) |
|  | Maso Sh | 600 | 10 (2%) |
|  | Gishu | 593 | 10 (2%) |
|  | Sr Tejitu | 704 | 12 (2%) |
|  | Sr Shage | 418 | 7 (2%) |
|  | Sr Habiba | 775 | 13 (2%) |
|  | **Total** | **5,184** | **88 (2%)** |
| **Arada** | | | |
|  | Feyine K3 | 606 | 19 (3%) |
|  | Demitu K4&7 | 665 | 21 (3%) |
|  | Zewditu K3&8 | 759 | 23 (3%) |
|  | Kedija K1&2 | 384 | 12 (3%) |
|  | Maryema K5&6 | 754 | 23 (3%) |
|  | **Total** | **3,168** | **98 (3%)** |
| **Awasho** | | | |
|  | Zenebech K9 | 460 | 13 (3%) |
|  | Zenebech Taffa K7 | 933 | 26 (3%) |
|  | Radia K4 | 465 | 13 (3%) |
|  | Bushe K10 | 460 | 13 (3%) |
|  | Mekiya | 700 | 21 (3%) |
|  | Tayiba Adem K9 | 168 | 5 (3%) |
|  | Sr Urji K2 | 454 | 13 (3%) |
|  | **Total** | **3,640** | **104 (3%)** |
| **Grand Total** | | **16,205** | **424 (3%)** |

Footnote: ^1^ PHCU: Primary Healthcare Units. ^2^ HHs: Households

**Supplementary Table 1-2. Household selection for community survey in Shashemene Woreda**

| **Name of PHCU^1^ and Kebeles** | | **Number of HHs^2^ in 2021** | **Number (%) of HHs^2^ selected** |
| --- | --- | --- | --- |
| **Faji Gole PHCU** | | | |
|  | Fiji Goba | 1,775 | 23 (1%) |
|  | Bute Filichaa | 1,683 | 22 (1%) |
|  | Faji Golee | 1,994 | 26 (1%) |
|  | Chefa Gutaa | 963 | 12 (1%) |
|  | Kubii Gutaa | 1,070 | 14 (1%) |
|  | Alleli Illuu | 1,729 | 22 (1%) |
|  | **Total** | **9,215** | **119 (1%)** |
| **Harbate PHCU** | | | |
|  | A/Harabaatee | 1,981 | 26 (1%) |
|  | E/Burqaa | 2,157 | 28 (1%) |
|  | Awaashoo | 2,079 | 27 (1%) |
|  | Abaaroo | 2,322 | 30 (1%) |
|  | Ebichaa | 1,222 | 16 (1%) |
|  | **Total** | **9,760** | **126 (1%)** |
| **Toga PHCU** | | | |
|  | Q/Borojjoota | 1,088 | 14 (1%) |
|  | B/Dannabaa | 1,332 | 17 (1%) |
|  | M/Dammaa | 1,494 | 19 (1%) |
|  | Togaa | 693 | 9 (1%) |
|  | D/Calalaqa | 873 | 11 (1%) |
|  | **Total** | **5,480** | **71 (1%)** |
| **Chabi PHCU** | | | |
|  | Chabi Dida Gnata | 1,462 | 19 (1%) |
|  | Tatesa Dedesa | 1,563 | 20 (1%) |
|  | Bura Borema | 1,451 | 19 (1%) |
|  | Kore Rogicha | 1,319 | 17 (1%) |
|  | Chulule Habera | 1,030 | 13 (1%) |
|  | Oine Chefo Umbure | 2,513 | 32 (1%) |
|  | **Total** | **9,339** | **120 (1%)** |
| **Grand Total** | | **33,795** | **436 (1%)** |

Footnote: ^1^ PHCU: Primary Healthcare Units. ^2^ HHs: Households

**Supplementary Table 2. Survey questionnaire structure and variables**

| **Survey questionnaire structure** | | **Variables** |
| --- | --- | --- |
| Form 1: General household information | Part A: Demographic data/General household information | - Household location - Respondent: age and sex and respondent’s relationship to this household - Household members: total number, age and sex of household members |
|  | Part B:  Socio-economic and education data | - Education - Occupation/income - Household asset - Housing/dwelling - Farm/domestic animals |
| Form 2:  KAP on cholera and cholera risk factors and prevention | Part A:  WaSH factors | **Water:**   - Main water supply for household for drinking, cooking/preparing food/meals, bathing, and cleaning - Distance of main water supply of household for drinking, cooking/preparing food/meals, bathing, and cleaning - Treatment of water for drinking, cooking/preparing food/meals, bathing, and cleaning prior to consumption/usage - Storing drinking water in household - Shared drinking water supply   **Waste disposal**   - Knowledge on proper disposal of household waste - Practice on household waste disposal - Distance of waste disposal site from household - Distance between main water supply of household to waste disposal site   **Toilet facility**   - Type of toilet facility available for household - Distance of toilet facility from household - Practice on utilisation of toilet facility - Practice on hand washing before and after urination/defecation   **Kitchen/Food handling**   - Availability of kitchen for cooking/preparing food/meals in household - Energy source of cooking/preparing food/meals - Cooking spot in household - Storing fresh/raw food items (e.g., vegetables, lettuce, fruits, dairy products, eggs, meat and fish) and left-over/prepared food/meals - Covering left-over/prepared food and reheating/recooking left-over/prepared food prior to consumption - Practice on hand washing prior to cooking/preparing food - Practice on food consumptions (e.g., cutlery, fingers) - Practice on sharing food (e.g., same pot) - Practice on hand washing (e.g., with soap, only water, rub hands with other things, etc.) - Source of main fresh/raw food items (e.g., self-farming, store, street vendor, family, etc.)   **WaSH practices related to raising animals/livestock**   - Keeping areas of animals/livestock and domestic animals/pets - Water supply for farm animals/livestock and domestic animals/pets - Practice on hand washing after feeding/watering and cleaning farm animals/livestock areas   **Crop farming**   - Practice on hand washing after fieldwork for crop farming |
|  | Part B:  Healthcare associated factors (such as vaccination status, disease perception) | **Vaccination status**   - Vaccines received for children under 6 years of age since birth: e.g., OCV (2 doses given 2 weeks apart), Rotavirus/RV (2/4/6months), Tuberculosis/BCG (at birth), Hepatitis B/HepB (at birth/1-2months/6-18months), Diphtheria/DTaP (2/4/6 months/15-18months/4-6years), Haemophilus influenzae b/Hib (2/4/6months/12-15months), Pneumococcus/PCV(2/4/6months/12-15months), Polio/IPV/OPV(2/4months/6-18months/4-6years), Influenza (yearly tarting from 6months), Measles-Mumps-Rubella/MMR(12-15months/4-6years), Varicella (12-15months/4-6years), Hepatitis A/HepA (12-23months), other vaccines   **Disease perception**   - Knowledge and perception towards causes and prevention of common infectious diseases including cholera |
| Form 3: healthseeking behaviour | Part A: Healthseeking behaviour and utilization | **Availability and accessibility to HCF**  **Healthseeking behaviour associated with symptoms of cholera**   - Where household adult/pediatric members *usually* seek healthcare (e.g., healthcare options: ECCP-HCF, other-HCF, physician, pharmacy, traditional healers, self-treatment, no-treatment)   **Healthcare utilization** **associated with suspected cholera, diarrhea and any illness**   - Where household adult/pediatric members *actually* sought healthcare in the last several years (e.g., healthcare options: ECCP-HCF, other-HCF, physician, pharmacy, traditional healers, self-treatment, no-treatment)   **Antibiotics use**   - Practice on antibiotics use as self-medication associated with suspected cholera, diarrhea, fever, respiratory illness, and other symptoms |

**Supplementary Table 3. Definition of study variables on water, sanitation, and hygiene^1^**

| Variable | Definition |
| --- | --- |
| Water | |
| At least basic^2^ | Drinking water from an improved source (i.e., piped water, borehole or tube wells), protected dug well, protected spring, rainwater, and packaged or delivered water and accessible on premises, available when needed within 30 minutes |
| Limited | Drinking water from an improved source provided that the collection time is more than 30 minutes for a round trip, including queuing |
| Unimproved | Drinking water from an unprotected dug well or unprotected spring |
| Surface water | Drinking water directly from a river, dam, lake, pond, stream, canal, or irrigation canal. |
| Sanitation | |
| At least basic^2^ | Use of improved facilities (i.e., flush/pour flush to the piped sewer system, septic tanks, or pit latrines; ventilated improved pit latrine, and composting toilets or pit latrines with slabs) that are not shared with other households |
| Limited | Use of improved facilities shared between two or more households |
| Unimproved | Use of pit latrines without a slab or platform, hanging latrines or bucket latrines |
| Open defecation | Disposal of human faeces in fields, forests, bushes, open bodies of water, beaches, and other open spaces or with solid waste |
| Hygiene | |
| Basic hygiene | Availability of a handwashing facility having both soap and water on the household premises. Handwashing facilities may be fixed or mobile and may include a sink with tap water, bucket with taps, tippy-taps, and jugs or basins designated for handwashing |
| Limited hygiene | Availability of a handwashing facility lacking soap and/or water at home |
| No hygiene facility | No handwashing facility at home |

**Footnote:**

^1^ The WaSH variables used in the analyses were based on the World Health Organization (WHO) and United Nations Children’s Fund (UNICEF) Joint Monitoring Program (JMP) 2022 indicators.

^2^ ‘Safely managed’ and ‘Basic’ in the JMP service ladders was combined as ‘at least basic’ for the analysis.

| **Improved** | Tap water (inside the house; private) |
| --- | --- |
|  | Tap water (outside the house i.e., back-/courtyard; private) |
|  | Tap water (outside the house; public/shared) |
|  | Standpipe/Piped water (inside the house; private) |
|  | Standpipe/Piped water (outside the house i.e., back-/ courtyard; private) |
|  | Standpipe/Piped water (outside the house; public/shared) |
|  | Water truck (public; shared) |
|  | Bottled water |
| **Unimproved** | Well/Borehole (open/uncovered/unprotected) |
|  | Spring water |
|  | Well/Borehole (covered/protected) |
| **Surface water** | Natural surface water (i.e., lake, pond, river, stream) |
|  | Man made surface water (i.e., canal, irrigation canal) |
|  | Rainwater |
| **Unknown** | Other with no specification |
|  | Don’t Know |
|  | No response |

**Supplementary Table 4a. Categorization of drinking water source type**

**Supplementary Table 4b. Categorization of distance to drinking water source from the house**

| **Accessible** | < 1m |
| --- | --- |
|  | 1 to < 5m |
|  | 5 to < 25m |
|  | 25 to < 50m |
|  | 50 to < 100m |
|  | 100 to < 500m |
|  | 500 to < 1,000m |
| **Non-accessible** | ≥ 1,000m |
| **Unknown** | Don’t know |
|  | No response |

**Supplementary Table 4c. Summary and combination to determine final water source status**

| **Water source status** | **Categorization from Table 2-1a & 2-1b** |
| --- | --- |
| **At least basic** | “Improved” water source (Table 2-1a) **AND** Accessible (Table 2-1b) |
| **Limited** | “Improved” water source (Table 2-1a) **AND** Non-accessible (Table 2-1b) |
| **Unimproved** | Unimproved (Table 2-1a) |
| **Surface water** | Surface water (Table 2-1a) |
| **Unknown** | One of response (Table 2-1a & 2-1b) is “Unknown” |

**Supplementary Table 4d. Categorization of toilet type**

| **Improved** | Flush toilet/Water closet (inside) |
| --- | --- |
|  | Non-flush latrine/pit latrine (inside) |
|  | Ventilated Improved Pit Latrine (KVIP, inside) |
|  | Composting toilet (inside) |
|  | Flush toilet/Water closet (outside) |
|  | Non-flush latrine/pit latrine (outside) |
|  | Ventilated Improved Pit Latrine (KVIP, outside) |
|  | Composting toilet (outside) |
| **Unimproved** | NO toilet facility, dig a hole |
|  | NO toilet facility, use a bucket |
| **Open defecation** | NO toilet facility, free range/open defecation |
| **Unknown** | Other with no specification |
|  | Don’t know |
|  | No response |

**Supplementary Table 4e. Categorization of toilet sharing status**

| **Unshared** | On the property of this household (private, not shared) |
| --- | --- |
| **Shared** | On the property of a neighboring household (private, shared) |
|  | In the community (public, shared) |
| **Unknown** | Other with no specification |
|  | Don’t know |
|  | No response |

**Supplementary Table 4f. Summary and combination to determine final sanitation status**

| **Sanitation status** | **Categorization from Table 2-2a & 2-2b** |
| --- | --- |
| **At least basic** | “Improved” facilities (Table 2-2a) **AND** “Unshared” (Table 2-2b) |
| **Limited** | “Improved” facilities (Table 2-2a) **AND** “Shared” (Table 2-2b) |
| **Unimproved** | Unimproved (Table 2-2a) |
| **Open defecation** | Open defection (Table 2-2a) |
| **Unknown** | One of response (Table 2-2a & 2-2b) is “Unknown” |

**Supplementary Table 4g. Categorization of handwashing practice**

| **Basic hygiene** | Wash hands with soap and water |
| --- | --- |
| **Limited hygiene** | Wash hands with water only |
|  | Rub hands clean with some leaves/straw/ grass |
|  | Rub hands clean with some sand |
|  | Rub hands clean with some ash |
|  | Rub hands clean with some cloth/fabric |
| **No hygiene facility** | Don’t clean, not needed/not dirty |
|  | Don’t clean; nothing to clean hands nearby/on hands |
| **Unknown** | Other with no specification |
|  | Don’t know |
|  | No response |

**Supplementary Table 4h. Summary and combination to determine final hygiene status**

| **Hygiene status** | **Categorization from Table 2-3** |
| --- | --- |
| **At least basic** | “Basic” hygiene (Table 2-3a) for **BOTH** situations |
| **Limited** | “Limited” hygiene (Table 2-3a) for **BOTH** situations  “Basic” **AND** “Limited” hygiene (Table 2-3a) for respective situation |
| **No hygiene facility** | “No hygiene facility” (Table 2-3a) for **BOTH** practices  “Limited” **AND** “No hygiene facility” (Table 2-3a) for respective situation |
| **Unknown** | One of response (to both situations) is “Unknown” |

**Figure legends**

**Supplementary Figure 1a. Map of Shashemene, West Arsi Zone, Oromia Region, Ethiopia**

**Supplementary Figure 1b. Map of Shashemene Town & Shashemene Woreda**

**Supplementary Figure 1c. Map of Shashemene Town**

**Supplementary Figure 1a** shows the location of Shashemene Town (ST) and Shashemene Woreda (SW) within West Arsi Zone located in Oromia region, Ethiopia. **Supplementary Figure 1b** exhibits the map of SW whereby the surveillance catchment area under the ECCP project highlighted in yellow and outside catchment area in green. ST in highlighted in orange is surrounded by SW area. **Supplementary Figure 1c** is the zoomed-in map of ST with ECCP surveillance catchment area marked in orange and outside catchment area in green.

**Supplementary Figure 2a. Geospatial map of surveyed households in SW**

**Supplementary Figure 2b. Geospatial map of surveyed households in ST**

**Supplementary Figure 2. Geospatial map of surveyed households**

The randomly selected households were interviewed for the community survey and pinpointed on this map with household GPS coordinates. **Supplementary Figure 2a** exhibits the SW households that participated in the survey pinpointed in blue bubble-dots within the ECCP surveillance catchment area in yellow. The rest of the SW that were outside of ECCP surveillance area are highlighted in green. ST is highlighted in orange. **Supplementary Figure 2b** exhibits the zoomed-in map of ST with ST households included in the survey pinpointed in red bubble-dots. Orange colored is the ECCP surveillance catchment area in ST. The remaining area in green shows the outside of ECCP surveillance catchment area in ST.

**Supplementary Figure 3. Percentage distribution of handwashing practices during critical times, Shashemene Town and Shashemene Woreda**

**Supplementary Figure 3** shows respondents’ hand-washing practice during the five critical times (before and after preparing foods, before and after visiting toilets and before eating) when handwashing is mandatory to prevent communicable diseases such as cholera. This was assessed based on the question that the respondent was asked whether she/he properly washes hands with soap/detergent and water during the specified times.

**Supplementary Figure 4. Geospatial map of cholera attack rate in study kebeles in Shashemene Town and Shashemene Woreda from 2016 to 2020**

**Supplementary Figure 4 a-e** shows the annual cholera attack rate per 10,000 population in 2016 **(4a)**, in 2017 **(4b)**, in 2018 **(4c)**, in 2019 **(4d)**, in 2020 **(4e)**; and the mean attack rate per 10,000 population from 2016 to 2020 **(4f)**. The bold line divides each cluster in Shashemene Woreda: from the top left, Chabi cluster, Faji Gole cluster, Toga cluster, Harabate cluster in a clockwise direction encircling Shashemene Town in the middle. Each kebele’s names is described on the corresponding kebele.

**Supplementary Figure 5. Annual trend of cholera attack rate by kebele, from 2016 to 2020**

**Supplementary Figure 5** shows the annual trend of cholera attack rate by kebele in the study catchment area, from 2016 to 2020. Kebeles at first row (i.e., Abosto, Arada, Alelu, and Awasho) are administered in Shashemene Town and other kebeles are nested in Shashemene Woreda.

**Supplementary Figure 6. Geospatial map of the proportion of surveyed households with ‘at least basic’ WaSH status in Shashemene Town and Shashemene Woreda, 2022**

**Figure 6a-c** shows the proportion of surveyed households with ‘at least basic’ water **(6a)**, sanitation **(6b)**; and hygiene **(6c)** at kebele-level. The bold line divides each cluster in Shashemene Woreda: from the top left, Chabi cluster, Faji Gole cluster, Toga cluster, Harabate cluster in a clockwise direction encircling Shashemene Town in the middle. Each kebele’s names is described on the corresponding kebele.
