# Supplementary figures and images for: Dissecting Water, Sanitation, and Hygiene (WaSH) to Assess Risk Factors for Cholera in Shashemene, Oromia Region, Ethiopia
Source: Clin Infect Dis. 2024 Jul 12;79(Suppl 1):S53–62. doi: 10.1093/cid/ciae274 (PMC11244206; doi:10.1093/cid/ciae274)

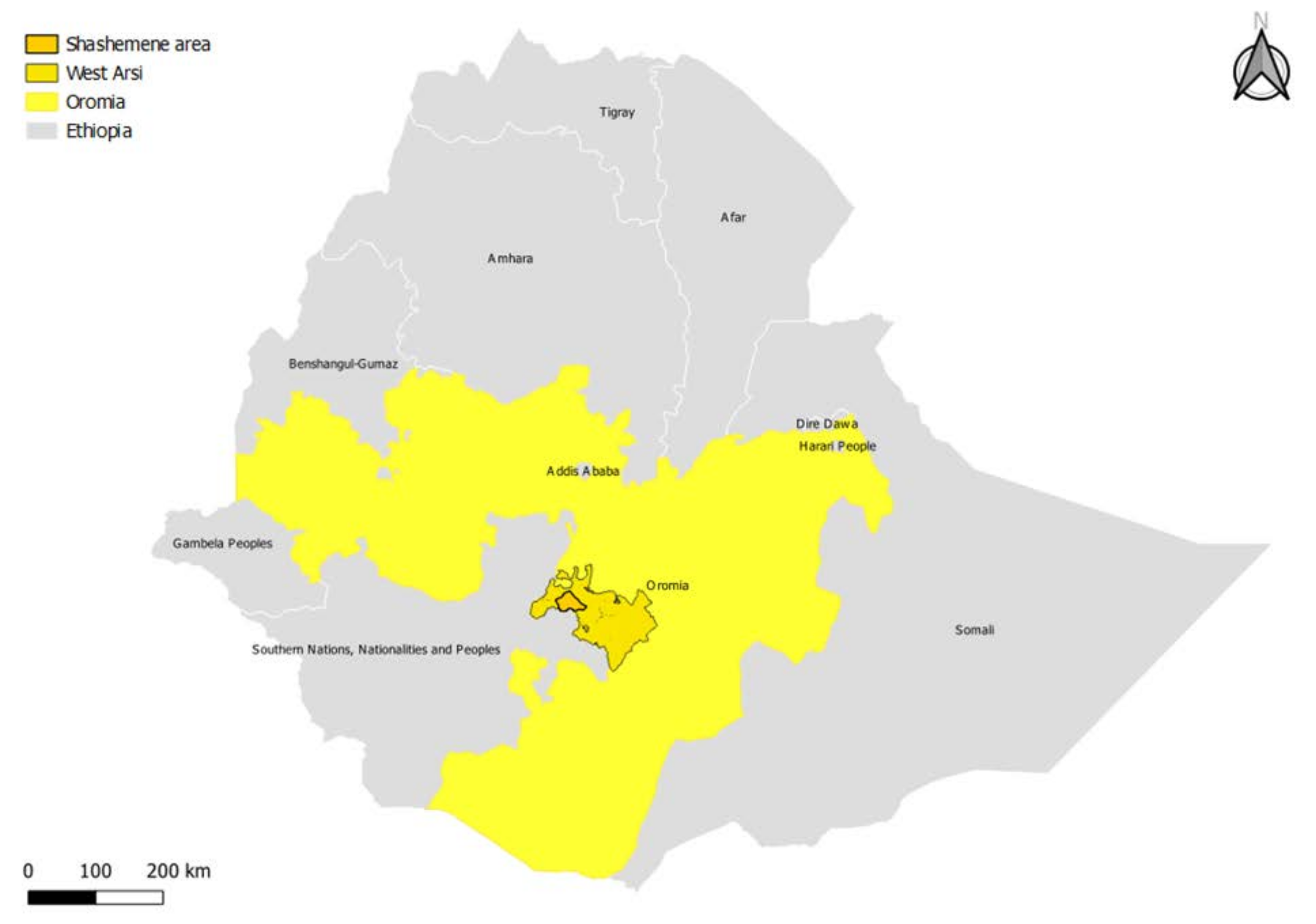

Supplement: ciae274_Supplementary_Data [file ciae274_supplementary_data.zip › SupplFig1a_Region_Ethiopia_29APR2024_x.tif]

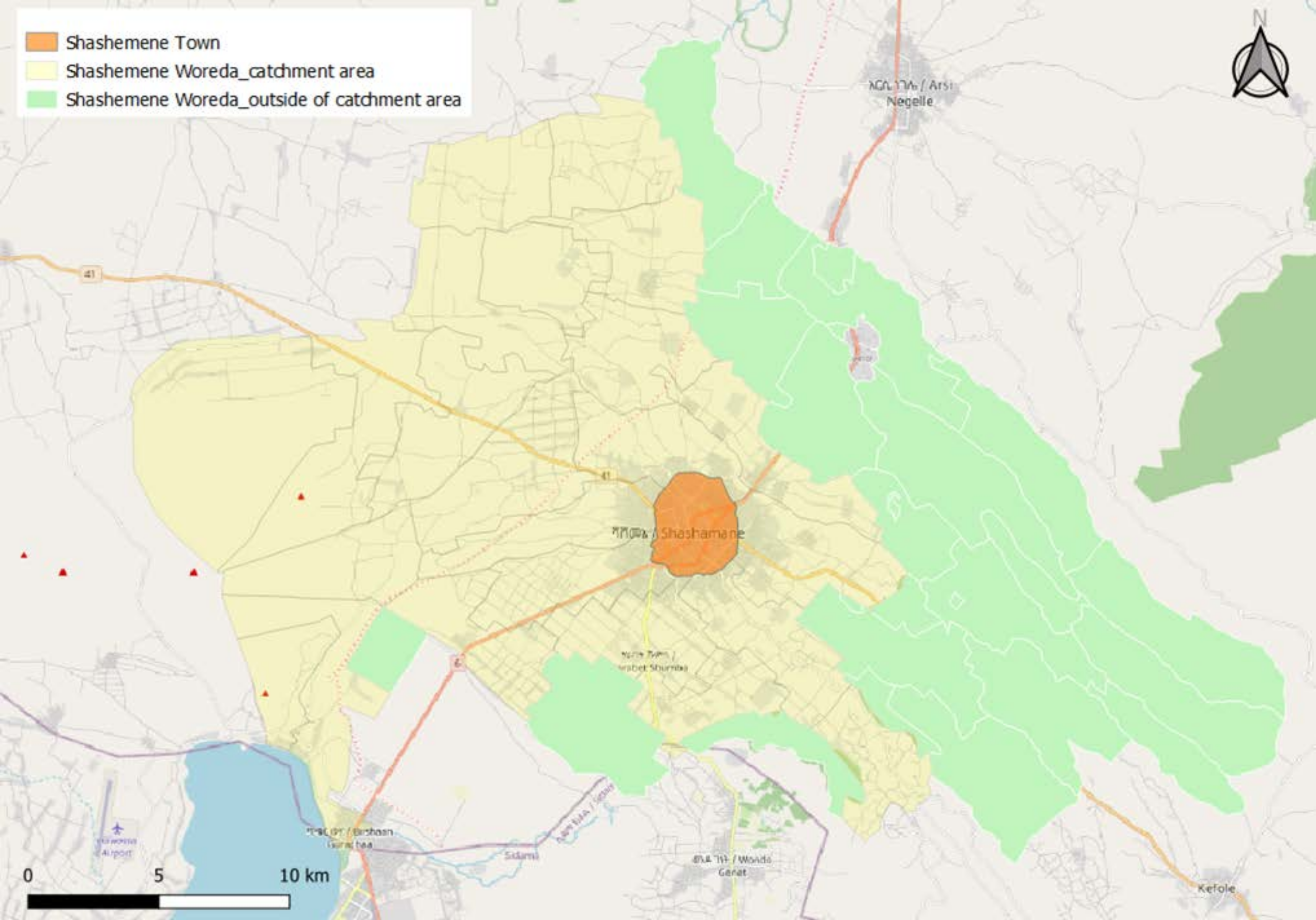

Supplement: ciae274_Supplementary_Data [file ciae274_supplementary_data.zip › SupplFig1b_28MAR2024_x.tif]

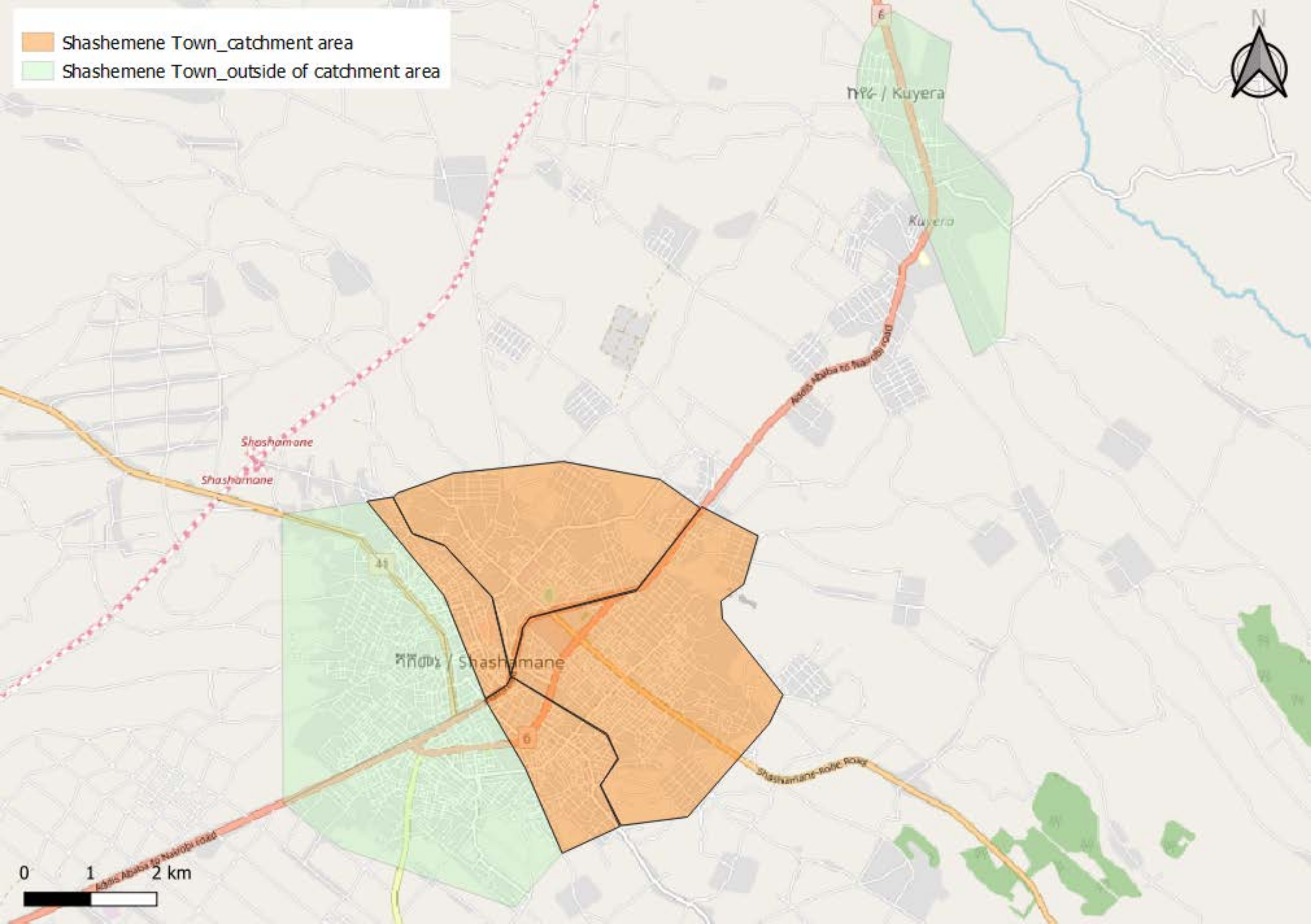

Supplement: ciae274_Supplementary_Data [file ciae274_supplementary_data.zip › SupplFig1c_Map of Shashemene Town_28MAR2024_x.tif]

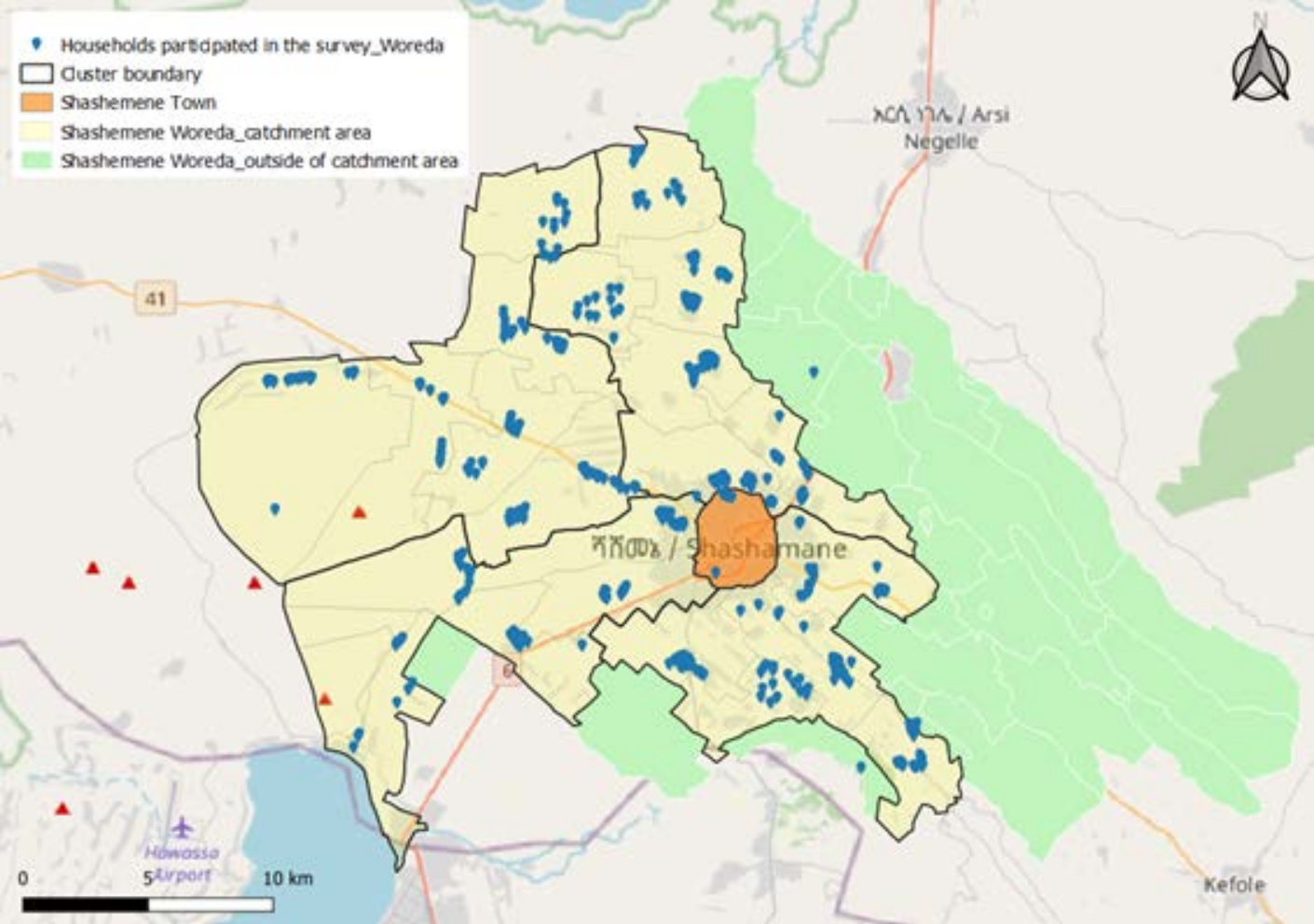

Supplement: ciae274_Supplementary_Data [file ciae274_supplementary_data.zip › SupplFig2a_Geospatial map SW_28MAR2024_x.tif]

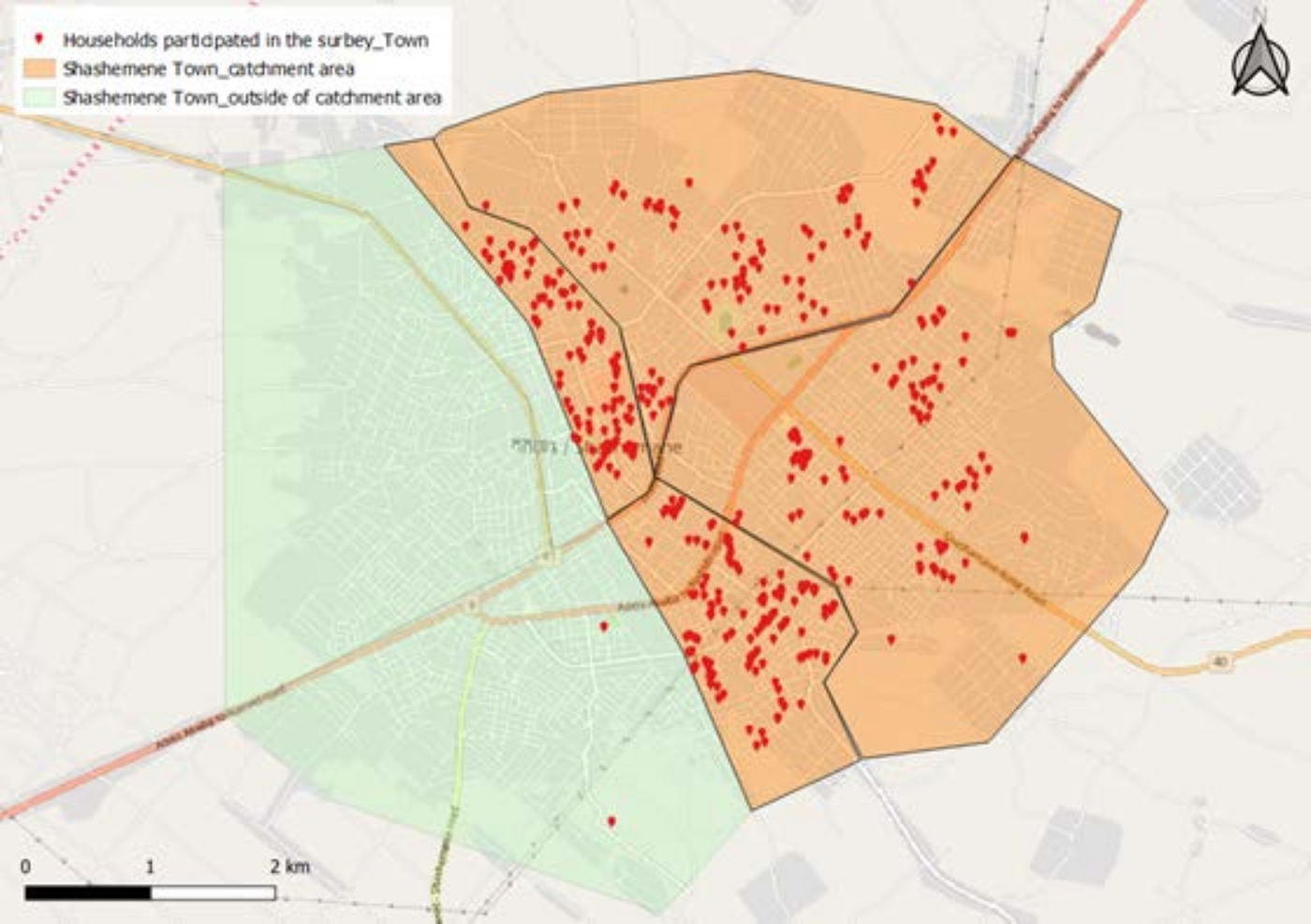

Supplement: ciae274_Supplementary_Data [file ciae274_supplementary_data.zip › SupplFig2b_ST_28MAR2024_x.tif]

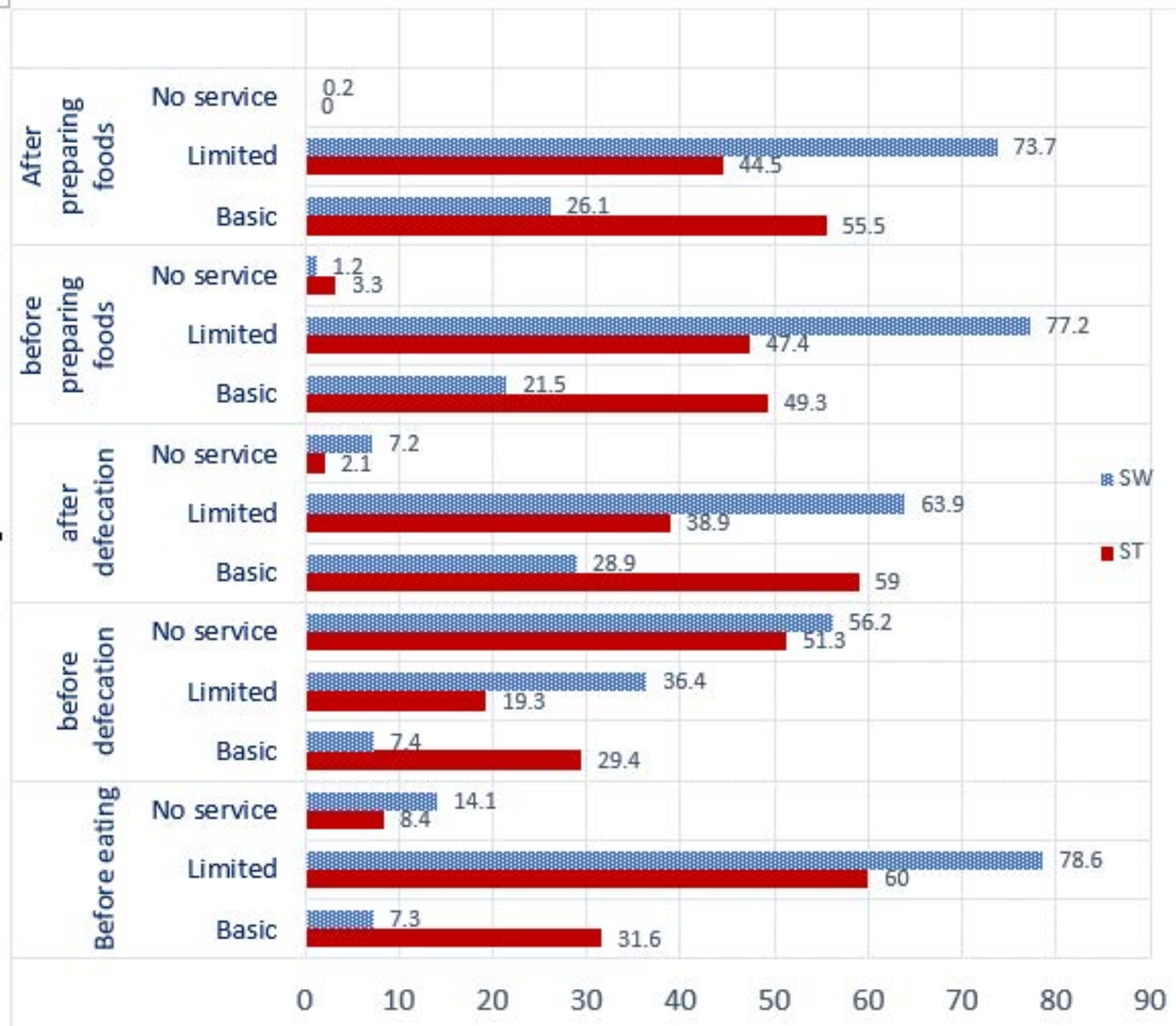

Supplement: ciae274_Supplementary_Data [file ciae274_supplementary_data.zip › SupplFig3_29APR2024_x.tif]

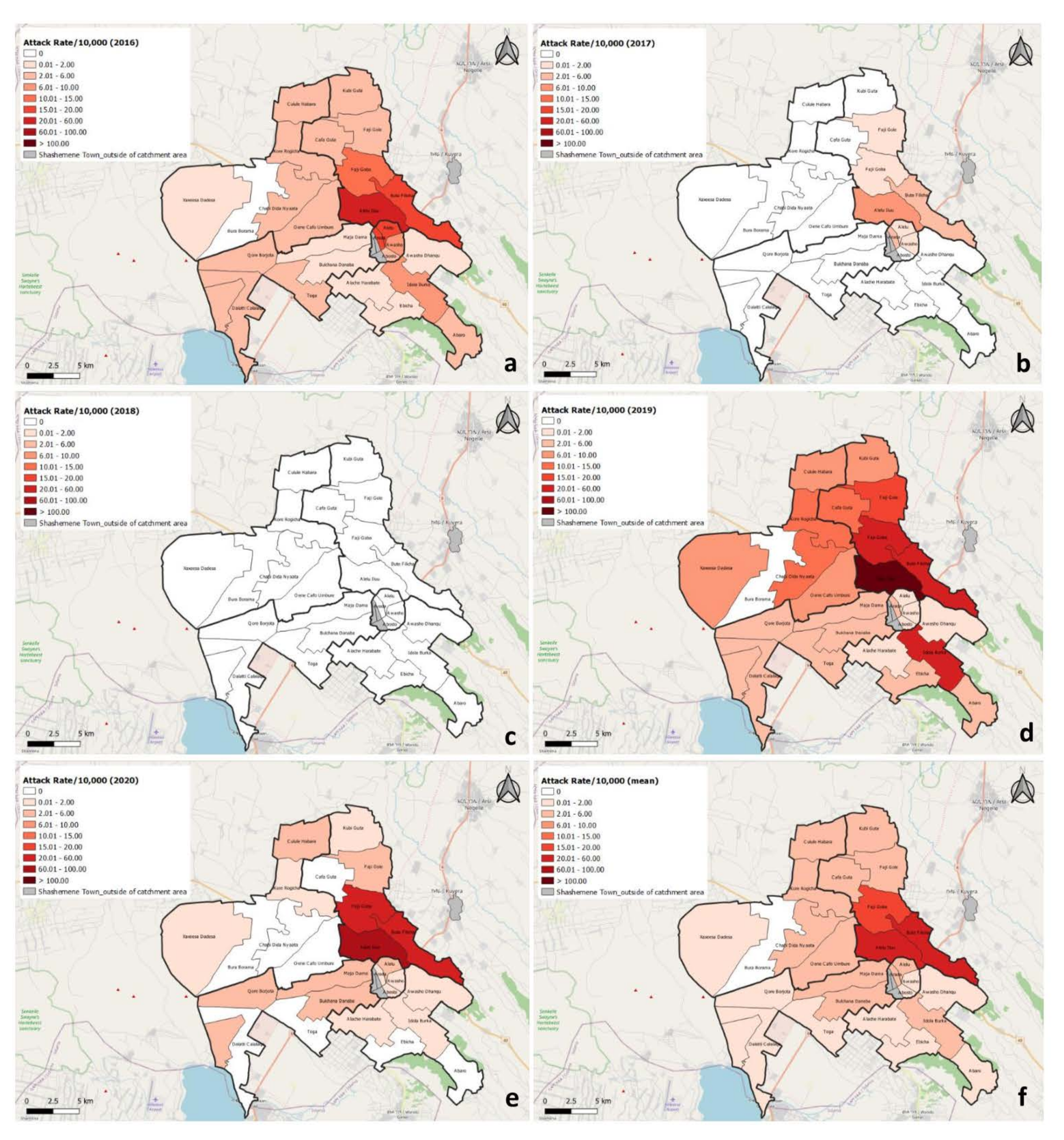

Supplement: ciae274_Supplementary_Data [file ciae274_supplementary_data.zip › SupplFig4_29APR2024_x.tif]

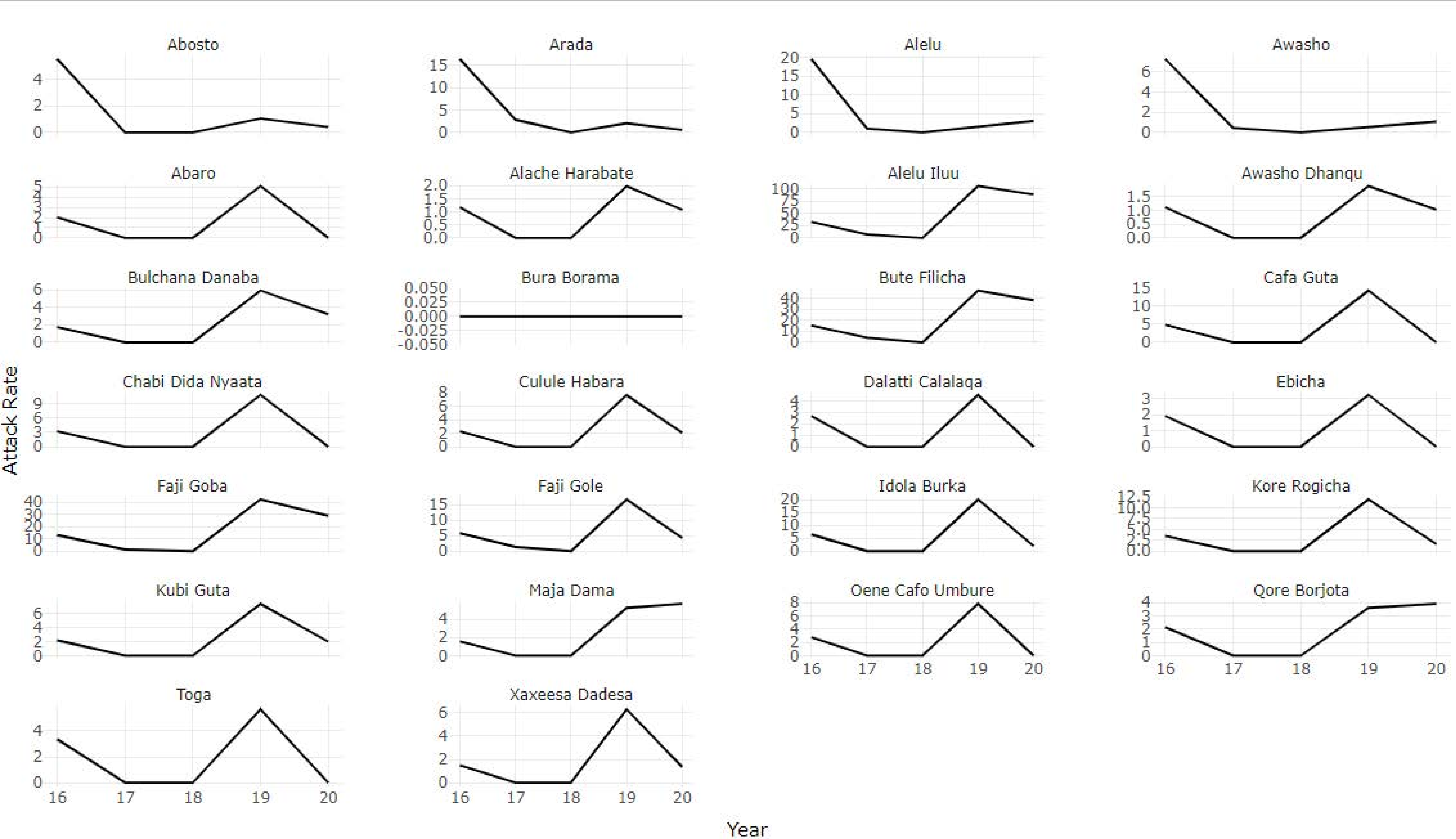

Supplement: ciae274_Supplementary_Data [file ciae274_supplementary_data.zip › SupplFig5_29APR2024_x.tif]

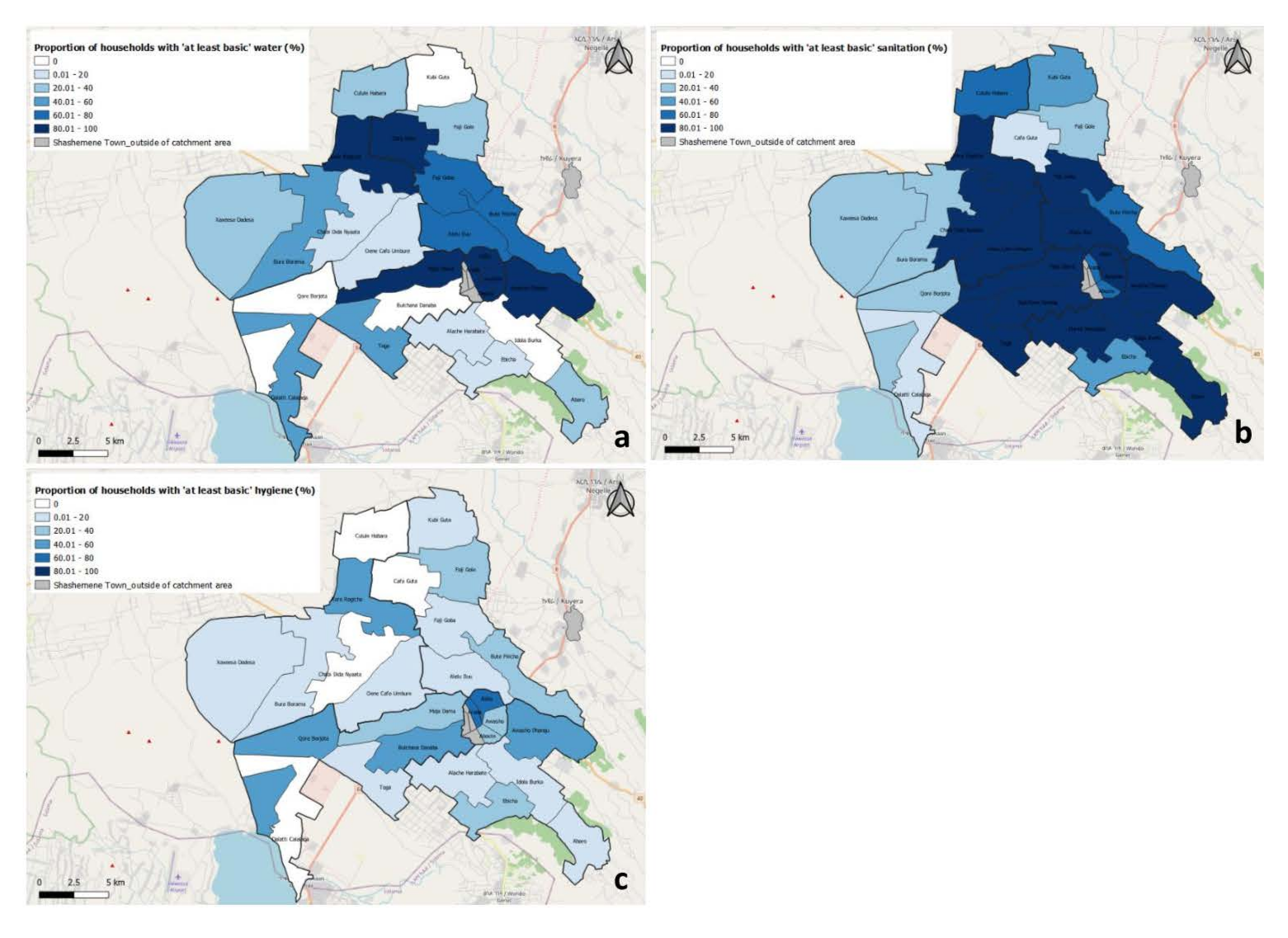

Supplement: ciae274_Supplementary_Data [file ciae274_supplementary_data.zip › SupplFig6_29APR2024_x.tif]
